# Brazilin Inhibits Growth and Induces Apoptosis in Human Glioblastoma Cells

**DOI:** 10.3390/molecules18022449

**Published:** 2013-02-21

**Authors:** Dae-Young Lee, Mi-Kyoung Lee, Geum-Soog Kim, Hyung-Jun Noh, Min-Ho Lee

**Affiliations:** 1 Herbal Crop Utilization Research Team, National Institute of Horticultural and Herbal Science, RDA, Eumseong 369-873, Korea; 2 Department of Neurosurgery, Ajou University School of Medicine, Suwon, 443-721, Korea; 3 Graduate School of Biotechnology and Department of Oriental Medicinal Materials & Processing, Kyung Hee University, Yongin 446-701, Korea

**Keywords:** apoptosis, brazilin, *Caesalpinia sappan*, glioma U87

## Abstract

Brazilin, isolated from the methanol extract of the heart wood of *Caesalpinia sappan*, sensitizes cancer cells to apoptosis. Glioblastoma multiforme (GBM), which accounts for most cases of central nervous system malignancy, has a very poor prognosis and lacks effective therapeutic interventions. We, therefore, investigated the effects of different concentrations of and different periods of exposure to brazilin on cell proliferation and apoptosis in the glioma U87 cell line. Cell proliferation was investigated by MTT assays and growth curve analysis, apoptosis was assessed by FACS analysis and western blot studies. Brazilin showed dose-dependent inhibition of cell proliferation and induction of apoptosis in glioma cells. It also increased the ratio of cleaved poly-(ADP)-ribose polymerase and decreased the expression of caspase-3 and caspase-7.

## 1. Introduction

Glioblastoma multiforme (GBM) is the most common and most malignant type of glioma [1]. It is an aggressive, invasive and difficult to treat primary brain tumor involving multiple genetic and chromosomal abnormalities [2] which make tumor cells proliferate uncontrollably and invade aggressively. Unfortunately, currently there exists a lack of effective therapeutic treatments for GBM. Standard therapy includes surgical resection, external beam radiation and chemotherapy, with no known curative therapy [3,4]. Therefore, it is critical to develop new and effective therapeutic agents that can inhibit the growth of human glioblastoma cells.

Sappan Lignum, the heart wood of *Caesalpinia sappan* L., is used traditionally for large number of ailments and reported to have a wide variety of medicinal properties. Its anti-inflammatory, anti-proliferative, and anti-oxidant activities have been reported [5,6,7]. Brazilin is a naturally occurring red pigment which is oxidized by air and light. Brazilin is a promising chemopreventive agent since it is generally non-toxic and interferes with the process of carcinogenesis. Several synthetic types brazilin analogues have demonstrated cancer-preventive properties towards a number of human cancer cell lines including HT29, A549, HL60, and K562 in MTT assays [8]. Tumorigenesis is a multistage process of accumulation of genetic alterations. Suppression of cell proliferation and induction of differentiation and apoptosis during the promotion and progression stages are ideal preventive targets. Recent studies indicate that glioblastoma cells are induced to undergo apoptosis and anticancer behaviors when treated with one of several natural or synthetic agents such as curcumin, jaceosidin, and sorafenib [9,10,11,12]. However, the potential anticancer properties of brazilin against glioblastoma cells are not well known. In the present study, we report on the isolation of brazilin (**1**) from the heart wood of *Caesalpinia sappan* and discuss the structural determination of this substance using extensive spectroscopic methods. We also investigate the effect of brazilin on the proliferation and apoptosis of GBM cells. Our findings indicate that brazilin-mediated apoptosis in glioblastoma cells is associated with activation of caspases.

## 2. Results and Discussion

### 2.1. Identification of Brazilin

**Figure 1:**
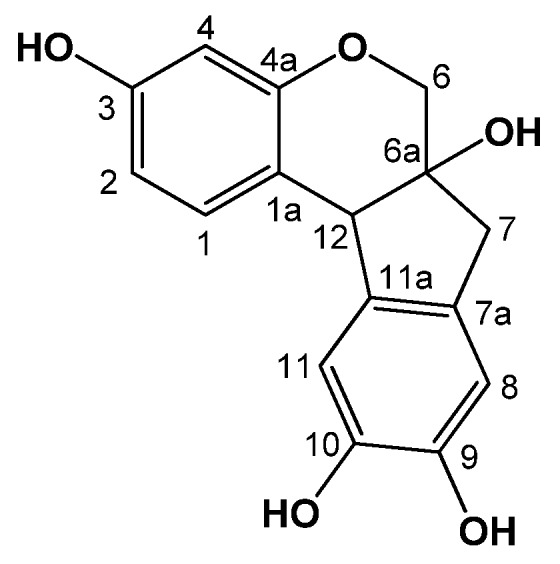
Chemical structure of compound **1**.

Compound **1** was obtained as reddish crystals from methanol, and exhibited a UV absorption maximum at 292 nm. The molecular formula was determined to be 286 [M]^+^ from the EIMS data. Comparisons of the NMR and MS data of the isolated compound **1** with reported values led to confirmation of the isolated compound as brazilin (**1**) [13] (Figure 1, Table 1).

**Table 1:** ^1^H- (400 MHz) and ^13^C-NMR (100 MHz) data of compound **1** (in CD_3_OD, *δ* in ppm, *J* in Hz)^a^.

No.	δ_H_	δ_C_	No.	δ_H_	δ_C_
1	7.17 (1H, d, *J* = 8.4 Hz)	137.3	7	3.07 (1H, d, *J* = 16 Hz, H-7a)	42.7
				2.74 (1H, d, *J* = 16 Hz, H-7b)	
1a		115.5	7a		131.3
2	6.47 (1H, dd, *J* = 8.4, 2.4 Hz)	109.9	8	6.60 (1H, s)	112.8
3	6.90 (1H, d, *J* = 3.2 Hz)	155.5	9		145.4
4	6.31 (1H, d, *J* = 2.4 Hz)	104.2	10		145.1
4a		157.6	11	6.71 (1H, s)	112.4
6	3.92 (1H, d, *J* = 11.2 Hz, H-6a)	70.7	12	3.96 (1H, s)	50.8
	3.68 (1H, d, *J* = 11.2, Hz, H-6b)				
6a		78.0			

^a^ Assignments were confirmed by DEPT, HSQC, and HMBC.

### 2.2. Brazilin-Induced Cytotoxicity in Human Glioblastoma U87 Cells

**Figure 2:**
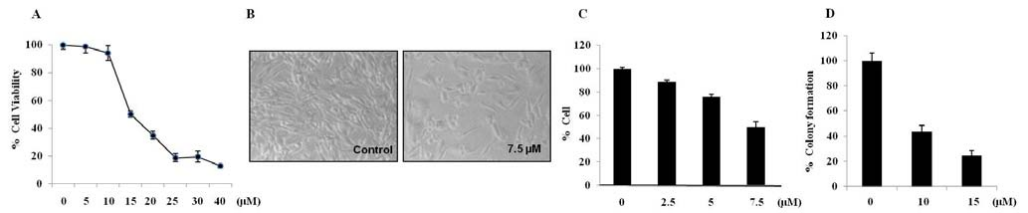
Brazilin inhibits glioblastoma cell growth; Morphological change, growth inhibition, and cytotoxicity in U87 cells. U87 cells were treated with 5, 10, 15, 20, 25, 30, or 40 μM of brazilin for 24 h, and cell viability was assessed by MTT assay (**A**). Morphological changes were observed using a microscope (magnification, 100×) (**B**). Effect of subtoxic concentrations of brazilin on proliferation of U87 cells. Cells were treated for 72 h with 2.5, 5, or 7.5 μM brazilin and stained with trypan blue for estimation of cell density (**C**). Incubation with brazilin for 14 days reduced clonogenic potential of U87 cells (**D**). All data are expressed as mean ± S.D. of triplicates.

In order to investigate whether brazilin treatments affected cell viability, U87 cells were treated with various concentrations (0–40 μM) of brazilin for 24 h. As shown in Figure 2A, treatments with brazilin induced cell death in U87 cells.

The morphological changes in brazilin-treated cells as seen by light microscopy are shown in Figure 2B. We observed both cell contraction and nuclear condensation in glioblastoma cells treated with brazilin. Compared to the untreated cells, brazilin-treated U87 cells became elongated, fewer in number, and more disorganized. The extent of the changes in cell shape and density depended on brazilin concentration. Significant morphological alterations and cell detachment were observed in 15 μM brazilin-treated cells (data not shown). At low concentrations (≤7.5 µM), brazilin caused decreases in cell viability and cell density but did not induce alteration in cell morphology (Figure 2B). This indicates that there exists a cytostatic or antiproliferative effect of brazilin at noncytotoxic concentrations. Therefore, we decided to test whether long-term exposure to subtoxic brazilin could decrease cell proliferation. We found that 7.5 µM of brazilin decreased cell proliferation by up to 50.2% ± 4.2% after 72 h exposure compared to untreated cells (Figure 2C). Brazilin-induced growth inhibition was independent of cell anchorage to extracellular matrix as evaluated by soft agar growth experiments. Results from clonogenic survival assays showed that brazilin treatment significantly decreased the clonogenic proliferation in U87 cells after 14 days (Figure 2D).

### 2.3. Brazilin-Induced High Accumulation of Sub-G1 Phase Cells

**Figure 3:**
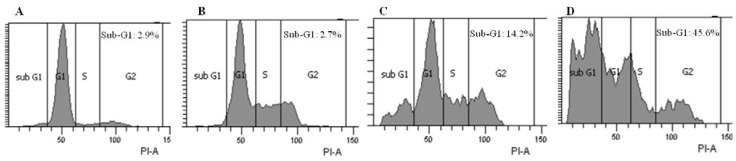
Brazilin induced high accumulation of sub-G1 phase cells. U87 cells were treated with no brazilin (**A**), 10 µM (**B**), 15 µM (**C**) or 20 µM (**D**) for 24 h. Cells were stained with PI, and DNA contents were analyzed with flow cytometry.

The inhibition of cell growth could be due to apoptosis mediated by cell cycle arrest. Since brazilin significantly induced cell death in U87, we examined whether brazilin affected the cell cycle, specifically. As shown in Figure 3, brazilin treatments increased the population of the sub-G1 phase cells compared with the control.

### 2.4. Brazilin Induced Activation of Caspase-3 and Caspase-7 and Cleavage of PARP

**Figure 4:**
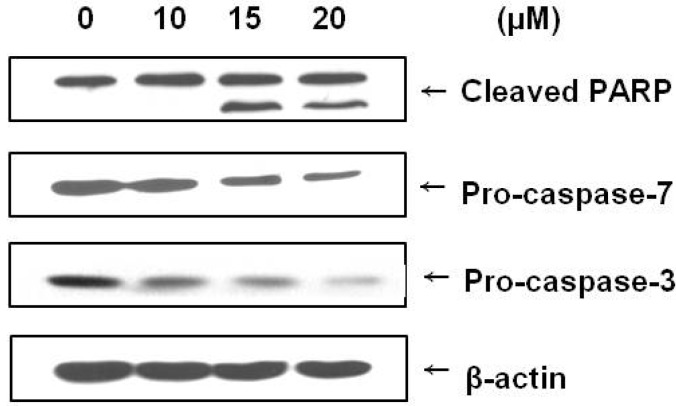
Immunoblot demonstrating the effect of brazilin treatment on poly(ADP-ribose) polymerase (PARP) and caspases (3 and 7) in human U87 glioblastoma cells. Cells were incubated with different concentrations of brazilin for 24 h, lysed, and cellular proteins were separated by 12% SDS-polyacrylamide gels and transferred onto PVDF membranes. The membranes were probed with antiPARP, anticaspase-3, and anticaspase-7 antibodies. Proteins were visualized by the ECL detection system. Actin was used as an internal control.

Caspases are known to be important mediators of apoptosis and to contribute to overall apoptotic morphology by cleaving various cellular substrates. Since brazilin treatment induced apoptosis in U87 cells, we investigated whether this phenomenon was the result of the regulation of caspases and PARP cleavage. Caspase activation and PARP cleavage were evaluated using a Western blot analysis. As shown in Figure 4, brazilin treatment increased cleavage of PARP and decreased expression of procaspase-3 and procaspase-7. The immunoblotting data showed a decrease in procaspase-3, demonstrating the participation of caspase-3 in brazilin-induced apoptosis.

### 2.5. Discussion

Brazilin is known to inhibit the growth of cancer cells and to exhibit certain biological effects [14], but the mechanisms of these actions are still only partially understood. Glioblastomas usually survive when there exist defects occurring in the expression and function of elements of the apoptotic machinery. Consequently, the identification of novel agents capable of triggering apoptosis in this type of tumor has become an important therapeutic objective [15]. The present study was undertaken to shed more light on the mechanisms by which brazilin exerts its anti-proliferative properties in cultured human glioblastoma cells. For the first time, we have demonstrated that brazilin inhibits the growth and induces apoptosis of human glioblastoma U87 cells.

Apoptosis is important not only during development and tissue homeostasis, but also in the pathogenensis of a variety of human disorders [16]. In this study, we observed that treatment with brazilin significantly inhibited U87 cell viability in a dose-dependent manner. The morphologic change of the cells also demonstrated that brazilin induced cell death, and we confirmed that brazilin increased the proportion of U87 glioblastoma cells in the sub-G1 portion of the cell cycle. These results indicate that brazilin is associated with the induction of apoptosis in U87 cells. The apoptotic mechanism has been extensively studied, and activation of caspase-3 and caspase-7 has been shown to occur in apoptosis [17]. We found that introduction of brazilin causes a decrease of procaspase-3 and procaspase-7 in human glioblastoma cells.

Activated caspase-3 induces PARP fragmentation [18], and PARP cleavage has been shown to be a hallmark of apoptosis. In this study, brazilin induced caspase-3, caspase-7, and PARP cleavage, indicating that treatment with brazilin induces apoptotic cell death in U87 cells through the activation of caspase-3 and subsequent cleavage of PARP. Therefore, we believe that the apoptotic mechanism of brazilin in brain cancer might occur at least partially through a caspase-dependent pathway.

## 3. Experimental

### 3.1. General

^1^H, ^13^C, and 2D NMR spectra were recorded on a Varian Unity Inova AS 400 FT-NMR instrument, and chemical shifts were given in δ (ppm) with reference to tetramethylsilane (TMS) used as an internal standard. Melting points were obtained using a Fisher-Johns Melting Point Apparatus equipped with a microscope. Ultraviolet spectra were measured on a Shimadzu model UV-1601 spectrophotometer. Optical rotations were measured on a JASCO P-1010 digital polarimeter. EIMS spectra was obtained using a JEOL JMS-700 mass spectrometer. Silica gel 60 (Merck, 230–400 mesh), LiChroprep RP-18 (Merck, 40–63 *μ*m), and Sephadex LH-20 (Amersham Pharmacia Biotech., Uppsala, Sweden) were used for column chromatography (C.C.). Pre-coated silica gel plates (Merck, Kieselgel 60 F_254_, 0.25 mm) and pre-coated RP-18 F_254s_ plates (Merck) were used for analytical thin-layer chromatography analyses. Spots were visualized by spraying with 10% aqueous H_2_SO_4_ solution followed by heating.

### 3.2. Plant Material

The heart wood of *Caesalpinia sappan* was purchased at an herbal drug store in Seoul, Korea, and identity was confirmed by Prof. Dae-Keun Kim, College of Pharmacy, Woo Suk University, Jeonju, Korea. A voucher specimen (OMRL-091024) was deposited at the Laboratory of Oriental Medicine Research, Kyung Hee University, Yongin, Korea.

### 3.3. Extraction and Isolation

The air-dried heart wood of *C. sappan* (5 kg) was powdered and extracted three times with 20 L of aqueous 80% MeOH at room temperature for 24 h. After concentration *in vacuo*, the MeOH extract (512 g) was suspended in H_2_O (2 L) and then partitioned successively with EtOAc (2 L × 3) followed by concentration to give the EtOAc fraction (E, 35 g). Fraction E was subjected to a silica gel C.C. (6.5 × 20 cm) using a gradient of CHCl_3_–MeOH–H_2_O (18:3:1 → 15:30:1 → 10:3:1 → 7:3:1, 1.3 L each) to yield 16 fractions (E1 to E16). Fraction E6 [3.8 g, Ve/Vt 0.34-0.42] was subjected to silica gel C.C. [4 × 15 cm, CHCl_3_–MeOH (7:1, 5.5 L)] to give ten subfractions (E6-1 to E6-10). Subfraction E6-2 (537 mg, Ve/Vt 0.21-0.34) was separated by C.C. [RP-18 (4 × 6 cm), MeOH–H_2_O (1:1.5, 1.5 L)] to give compound **1** [85 mg, Ve/Vt 0.22-0.33, TLC (RP-18 F_254s_) *R_f_* 0.50, MeOH–H2O (1.5:1)].

### 3.4. Spectroscopic Data

*Brazilin* (**1**). Reddish crystals, Mp: 143–146 °C; [α]^25^_D_ + 76.5 (*c* = 0.5, MeOH). UV λ_max_ (MeOH) nm: 294; EIMS *m*/*z*: 286 [M]^+^; ^1^H- and ^13^C-NMR data, see Table 1.

### 3.5. Cell Culture

Propidium iodide (PI), 3-(4,5-dimethyl)-2,5-diphenyltetrazolium bromide (MTT), dimethyl sulfoxide (DMSO), and RNase A were purchased from Sigma Chemical Co. (St. Louis, MO, USA). Anti-PARP, anti-caspase-3 and anti-caspase-7 were from Cell Signaling Technology (Danvers, MA, USA). The human U87 malignant glioblastoma cell line was obtained from American Type Culture Collection (Rockville, MD, USA). The cells were grown and maintained in high-glucose Dulbecco’s Modified Eagle’s Medium (DMEM; Gibco BRL, Carlsbad, CA, USA) containing 1% penicillin/streptomycin and supplemented with 10% fetal bovine serum (FBS; Gibco BRL, Carlsbad, CA, USA). Cells were kept at 37 °C in a humidified atmosphere with 5% CO_2_.

### 3.6. MTT and Proliferation Assays

Cells were seeded on 96-well plates at a density of 1 × 10^4^ cells/well in 200 μL of medium. After 24 h of adhesion, the cells were treated with 0–40 μM of brazilin, and plates were incubated at 37 °C for 24 h. The number of living cells was determined by MTT assay. MTT was dissolved in PBS at a concentration of 5 mg/mL. From this stock solution, 10 μL per 100 μL of medium was added to each well, and plates were incubated at 37 °C for 4 h. Treatment of living cells with MTT produces a dark blue formazan product, whereas no such staining is observed in dead cells. The dark blue, crystal metabolized products of MTT were extracted by DMSO. Absorbance at 540 nm was determined and used for the measurement of the proportion of surviving cells. For the proliferation assay, cells in 6-well-plates were treated at 40–50% confluence for 72 h with a subtoxic concentration of less than 7.5 μM of brazilin. Three days after treatment, cells were collected and counted using trypan blue stain.

### 3.7. Morphological Observation

Cells were seeded on 24-well plates at a density of 5 × 10^4^ cells in 0.5 mL of medium. After 24 h of adhesion, the cells were treated with either 7.5 or 15 μM of brazilin, and the plates were incubated at 37 °C for 24 h. The morphological changes of the cells were observed with an inverted microscope.

### 3.8. Soft Agar Assay

U87 cells were plated in soft agar at a density of 10,000 cells/mL on a 6-well plate. For the base layer, 1.6% agar stock solution was melted in an autoclave, cooled to 40 °C in a water bath and then mixed with culture medium to obtain a solution of 0.8% agar in DMEM (2 mL) containing 10% FBS. Cell suspension placed on the top layer was comprised of DMEM (2 mL) with 10% FBS and 0.4% agarose in each well. Cells were successively plated into single cells and incubated for 2 weeks at 37 °C in humidified atmosphere of 5% CO_2_. U87 cells were then converted with 2 mL of new medium containing brazilin. Medium containing treatments were replaced every 72 h. At the end of 14 days, the number of colonies was counted.

### 3.9. Flow Cytometric Analysis

Cells were washed with phosphate buffered saline (PBS), trypsinized, and washed twice with ice-cold PBS by centrifugation at 800 g for 5 min. After an overnight incubation with 70% ethanol at −20 °C, the cells were washed twice with PBS. Then, final cell pellets were resuspended in 0.5 mL of propidium iodide (PI) solution (50 μg of PI, 4 mM of sodium citrate, 1 mg/mL of RNase A and 1% of Triton X-100) by gently vortexing. Cells were incubated for 30 min in the dark and then analyzed with FACSAria ^TM^III (Becton Dickinson, San Jose, CA, USA) using Cell Quest software.

### 3.10. Western Blot Analysis

Proteins (30 μg) were separated by sodium dodecyl sulfate polyacrylamide gel and electrotransferred onto PVDF membranes (Millipore, Bedford, MA, USA). Membranes were activated with methanol and incubated in TBS-T (20 mmol/L Tris-HCl, pH 7.5, 137 mmol/L NaCl, 0.05% Tween 20) containing 5% nonfat milk powder for 1 h at room temperature. Subsequently, the membranes were incubated for 12 h at 4 °C with the appropriate primary antibody (dilution range 1:1,000–1:2,000), rinsed with TBS-T and exposed to horseradish-peroxidase-linked anti-IgG antibodies for 1 h at room temperature. The proteins were visualized with chemiluminescence procedures (Amersharm Parmacia Biotech) according to the manufacturer’s instructions.

### 3.11. Statistical Analysis

The data shown are from three experiments and are presented as the mean ± S.D. The statistical significance between control (DMSO-treated) and brazilin-treated groups were carried out by means of Student’s *t* test. The results were considered significant at a *p*-value < 0.05.

## 4. Conclusions

We have demonstrated that brazilin isolated from *Caesalpinia sappan* is able to inhibit growth of U87 cells by inducing apoptosis at low concentrations, and the induction of apoptosis is associated with the caspase-related apoptotic pathway. Therefore, we speculate that the induction of apoptosis observed in this study may provide a distinct mechanism for the cancer therapeutic and chemo-preventive functions of brazilin. Further studies aimed at the determination of the therapeutic effects of brazilin on glioblastoma growth *in vivo* should provide interesting insights on the potential clinical usefulness of this agent in the treatment of brain tumors.

## Supplementary Materials

^1^H-NMR and ^13^C-NMR spectra of compound **1** are provided as supplementary materials, which can be accessed at: http://www.mdpi.com/1420-3049/18/2/2449/s1.
