# Supplementary material for: Brazilin Inhibits Growth and Induces Apoptosis in Human Glioblastoma Cells
Source: Molecules. 2013 Feb 21;18(2):2449–57. doi: 10.3390/molecules18022449 (PMC6270480; doi:10.3390/molecules18022449)

Supplementary Materials

In this 'Supporting Information' file for the manuscript "Brazilin Inhibits Growth and Induces Apoptosis in Human Glioblastoma Cells", ^{1}H-NMR, and ^{13}C-NMR spectra of compound **1** are available here as listed below.

Figure S1. ^{1}H-NMR of compound **1**.

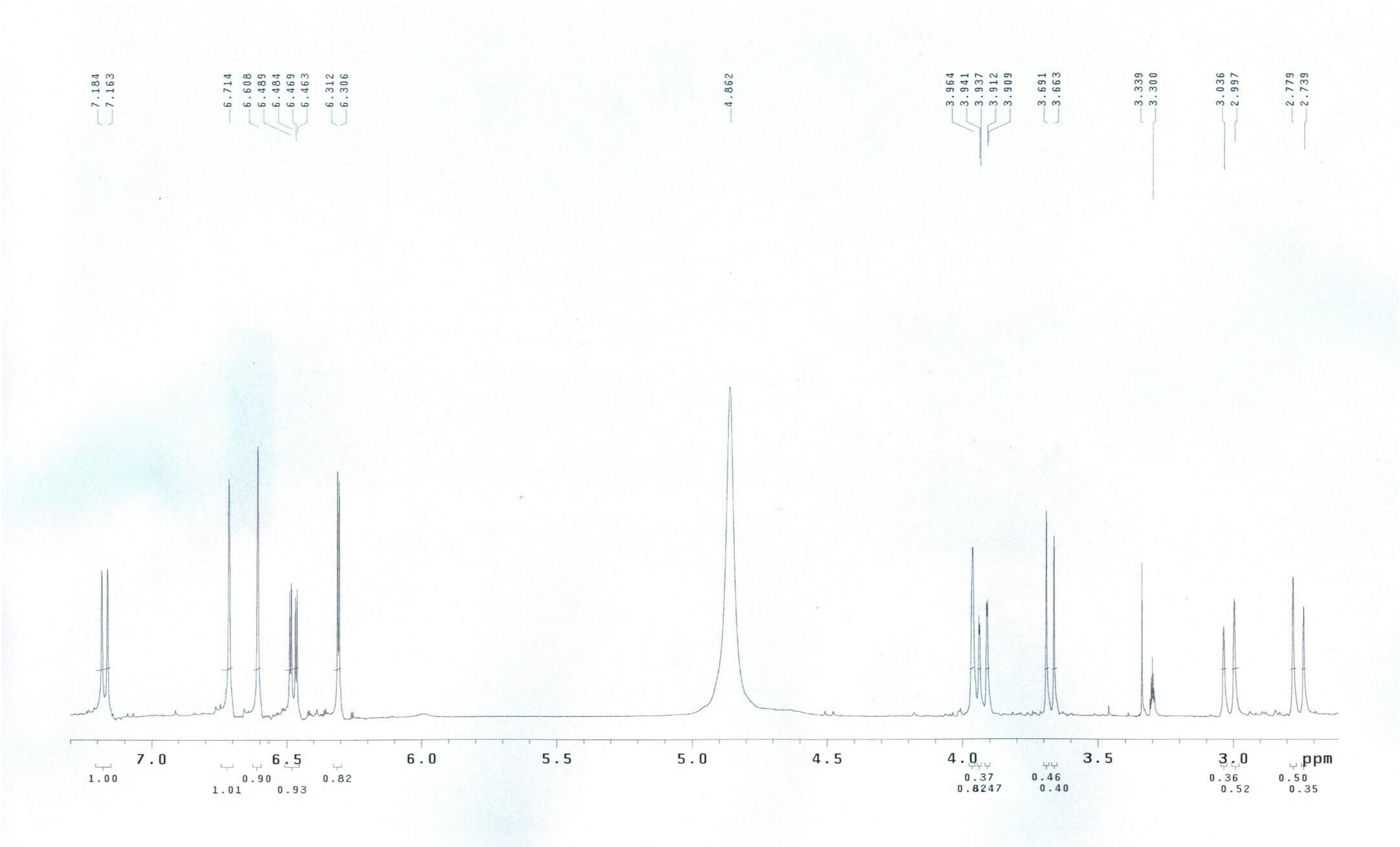

Figure S2. ^{13}C-NMR of compound **1**.

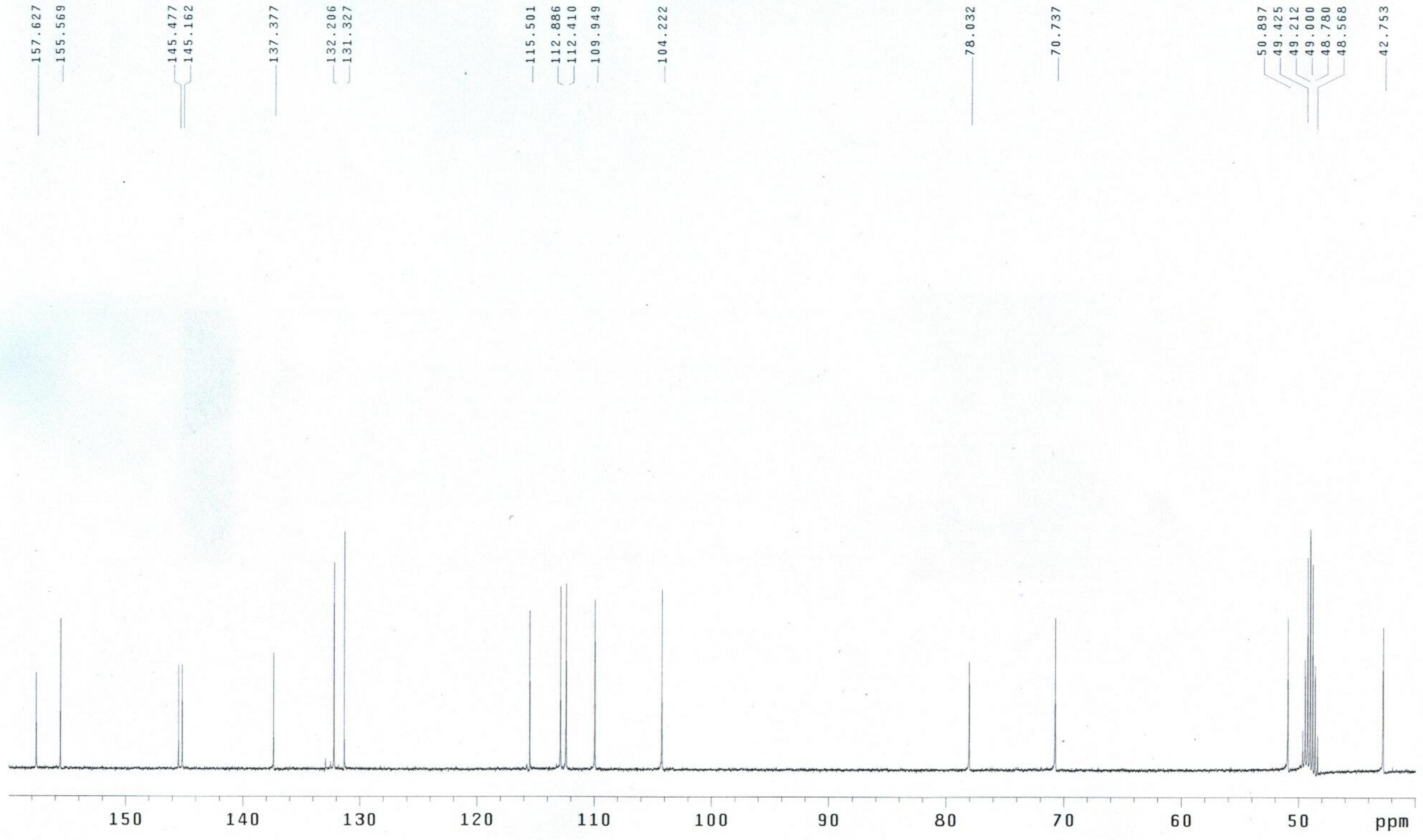

Supplement: Supplementary file 1 [file molecules-18-02449-s001.pdf]
